# Enhanced cell-permeant Cre protein for site-specific recombination in cultured cells

**DOI:** 10.1186/1472-6750-4-25

**Published:** 2004-10-22

**Authors:** Qing Lin, Daewoong Jo, Kassatihun D Gebre-Amlak, H Earl Ruley

**Affiliations:** 1 Department of Microbiology and Immunology, Room AA5206, MCN, Vanderbilt University, School of Medicine, 1161 21st Ave South, AA4210, Nashville, TN 37232-2363, USA

## Abstract

**Background:**

Cell-permeant Cre DNA site-specific recombinases provide an easily controlled means to regulate gene structure and function in living cells. Since recombination provides a stable and unambiguous record of protein uptake, the enzyme may also be used for quantitative studies of *cis*- and *trans*-acting factors that influence the delivery of proteins into cells.

**Results:**

In the present study, 11 recombinant fusion proteins were analyzed to characterize sequences and conditions that affect protein uptake and/or activity and to develop more active cell-permeant enzymes. We report that the native enzyme has a low, but intrinsic ability to enter cells. The most active Cre proteins tested contained either an N-terminal 6xHis tag and a nuclear localization sequence from SV40 large T antigen (HNC) or the HIV Tat transduction sequence and a C-terminal 6xHis tag (TCH_6_). The NLS and 6xHis elements separately enhanced the delivery of the HNC protein into cells; moreover, transduction sequences from fibroblast growth factor 4, HIV Tat or consisting of the (KFF)_3_K sequence were not required for efficient protein transduction and adversely affected enzyme solubility. Transduction of the HNC protein required 10 to 15 min for half-maximum uptake, was greatly decreased at 4°C and was inhibited by serum. Efficient recombination was observed in all cell types tested (a T-cell line, NIH3T3, Cos7, murine ES cells, and primary splenocytes), and did not require localization of the enzyme to the nucleus.

**Conclusions:**

The effects of different sequences on the delivery and/or activity of Cre in cultured cells could not be predicted in advance. Consequently, the process of developing more active cell-permeant recombinases was largely empirical. The HNC protein, with an excellent combination of activity, solubility and yield, will enhance the use of cell-permeant Cre proteins to regulate gene structure and function in living cells.

## Background

The Cre recombinase from bacteriophage P1 has been widely used to induce DNA sequence-specific recombination in mammalian cells [1]. The enzyme, which catalyzes recombination between 34 nucleotide LoxP sequences during P1 genome replication, has been used in a variety of genetic applications to regulate gene structure and function. These include conditional mutagenesis, gene replacement, chromosome engineering, and regulated gene expression [2-4]. However, the use of site-specific recombination in genetic studies is frequently hampered by difficulties expressing the recombinase in cells at the desired time and place [5]. Moreover, the use of Cre expression vectors is constrained by the fact that prolonged exposure to the enzyme can be lethal to cells [4,6,7].

To address these problems, we [8] and others [9-12] have developed membrane-permeable Cre recombinase proteins that are capable of entering cells by a process of protein transduction. Protein transduction exploits properties of specific protein sequences [termed protein transduction domains (PTDs)] that enhance the delivery of macromolecules – including peptides, proteins, and DNA fragments – into living cells [13-15]. Cell-permeant Cre proteins provide an effective means to regulate gene structure and function in living cells, and Cre-mediated recombination provides a potentially useful reporter system with which to study the process of protein transduction itself. In particular, recombination provides a stable and quantitative record of protein uptake that circumvents problems of distinguishing between internalized and cell-associated proteins [16].

In our previous report, recombinant fusion proteins bearing the 12 amino acid membrane translocation sequence (MTS) from fibroblast growth factor 4 (FGF-4) were used to deliver enzymatically active Cre proteins directly into mammalian cells. Of the four recombinant proteins tested, an enzyme containing an N-terminal 6xHis affinity tag, a nuclear localization sequence (NLS) from SV40 large T antigen, and the FGF-4 MTS (HNCM), displayed the best combination of yield, solubility, nuclear localization and enzymatic activity within cells. Recombination was observed in greater than 70% of cells treated with 10 μM HNCM for 2 hours. Widespread recombination was also observed in mice following intraperitoneal administration of HNCM, indicating that a wide variety of terminally differentiated cell types can internalize cell-permeant Cre and are competent to undergo site-specific recombination.

In the present study, eleven recombinant Cre proteins were prepared in order to evaluate sequences affecting the uptake and/or activity of the enzyme in cultured cells and if possible to develop more active recombinases. Several constructs were designed to compare the activities of different PTDs, including the FGF-4 MTS [17], sequences from HIV TAT [18] and a (KFF)_3_K sequence that was previously used to deliver peptide nucleic acid (PNA) conjugates into cells [19]. Only the Tat sequence promoted the delivery of active Cre into cells, while all three PTD sequences adversely affected the solubility of recombinant proteins containing polyhistidine tags. The contribution of the SV40 large T antigen NLS [20] was also examined to understand apparent differences in the behaviour of cell-permeant Cre and proteins expressed following gene transfer. Thus, the activity of cell-permeant Cre was enhanced by the SV40 large T antigen NLS [8,10], whereas, the native Cre protein appears to possesses a functional NLS, whose activity was not augmented by the T antigen NLS [21]. We report that polyhistidine tags (6xHis) frequently used for protein affinity purification [22] and the large T antigen NLS each separately enhance cellular uptake of enzymatically active Cre, and we describe the development of a cell-permeant Cre recombinase with an excellent combination of activity, solubility and ease of purification.

## Results

### Recombinant Cre fusion proteins

**Figure 1:**
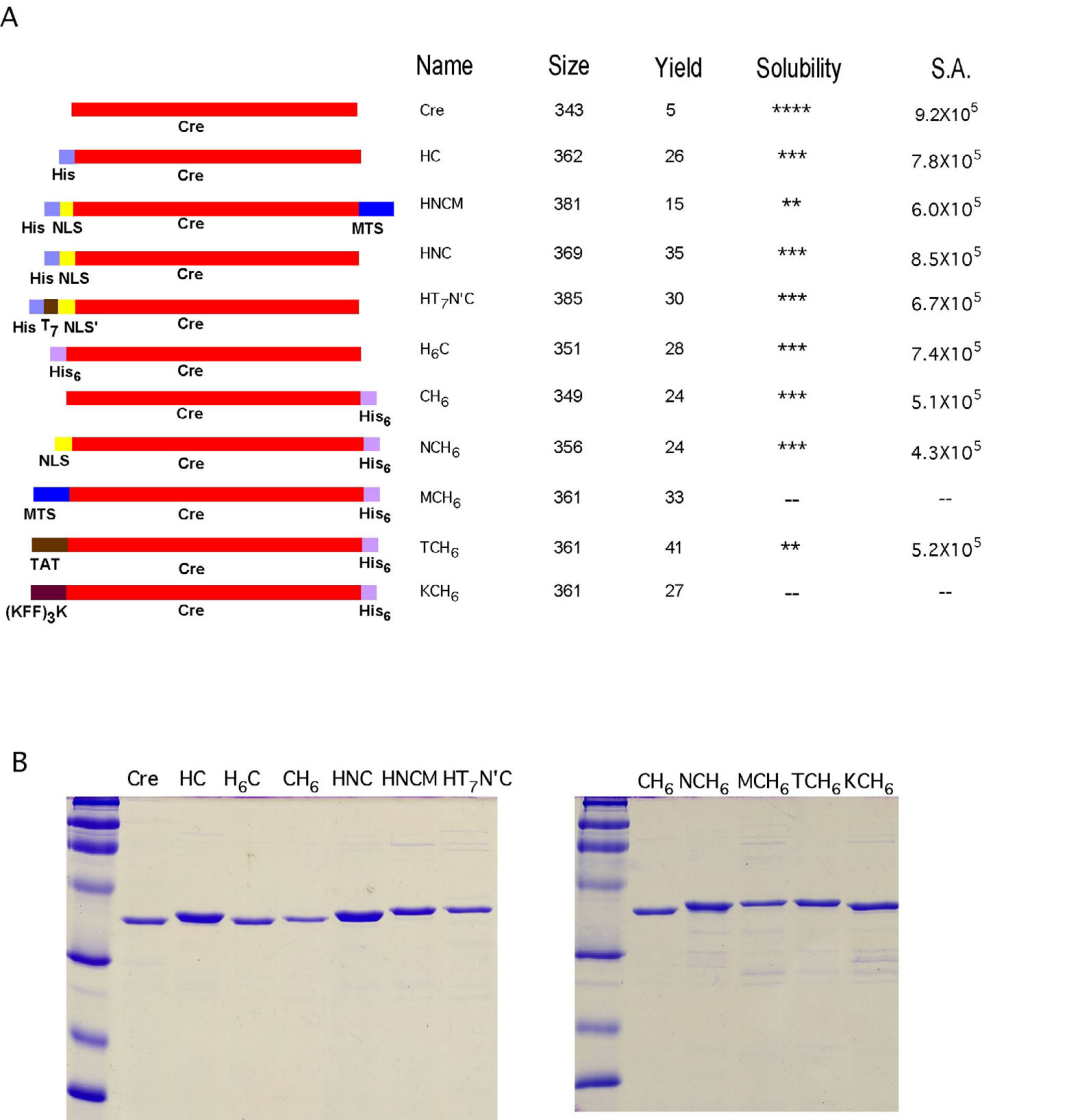
**Recombinant Cre fusion proteins. **(A) Structures of recombinant Cre fusion proteins. Cre sequences from nucleotide 484 to 1513 (GenBank X03453) were expressed unaltered or incorporated into fusion proteins containing one or more of the following elements: His (MGSSHHHHHHSSLVPRGSH); His_6 _(MHHHHHH or HHHHHH for N- and C-terminal sequences, respectively); NLS (PKKKRKV); NLS' (PKKKKKV); T_7 _(MASMTGGQQMG); MTS (AAVLLPVLLAP); TAT (YGRKKRRQRRR) and (KFF)_3_K (KFFKFFKFFK). The table on the right lists the name, size (number of amino acids), yield following purification from *E. coli *(mg/L), relative solubility, and in vitro specific activity (units per milligram of enzyme) of each fusion protein. (B). Analysis of purified Cre fusion proteins. Purified Cre fusion proteins were fractionated by SDS-PAGE and stained with Coomassie blue.

Eleven recombinant Cre proteins were prepared in order to evaluate sequences affecting the uptake and/or activity of cell-permeant enzymes (Figure 1A). Native Cre (Cre) corresponds to the protein encoded by the P1 phage genome [23]. HC and H_6_C have amino-terminal hexahistidine tags consisting of MGSSHHHHHHSSLVPRGSH and MHHHHHH, respectively, while CH_6 _is similar to H_6_C except the His tag is on the C-terminus. His-NLS-Cre (HNC) is similar to HC except a nuclear localization sequence (PKKKRKV) from SV40 large T antigen [20] is positioned between His and Cre. HT_7_N'C is similar to HNC except the His tag contains 11 additional amino acids (MASMTGGQQMG) from the pET28a(+) polylinker and the arginine of the NLS sequence was converted to a lysine. This change, which resulted from an altered PCR primer, is unlikely to affect nuclear localization activity. HNCM, described previously [8], contains a membrane translocation sequence (MTS) from the leader sequence of FGF-4 positioned at the C-terminus of HNC. Finally, four different sequences, each reported to have protein transduction activity [17,19,24], were placed on the amino terminus of CH_6_. These consisted of the FGF-4 MTS (MCH_6_), an SV40 large T antigen nuclear localization sequence (NCH_6_), the HIV Tat sequence (TCH_6_), and a (KFF)_3_K sequence (KCH_6_).

Cre proteins were expressed from pET28a(+) plasmids in *E. coli *and, except for native Cre, were purified by Ni^2+ ^affinity chromatography under non-denaturing conditions [8]. The native enzyme was purified by a combination of hydroxyapatite column chromatography and Sephacryl S-100 HR FPLC. All proteins were expressed at high levels, with yields of purified proteins ranging from 5 to 41 mg/L of *E. coli *culture (Figure 1). All of the enzymes except TCH_6 _and KCH_6 _could be dialyzed against DMEM or RPMI media and stored at -20°C until use. However, TCH_6 _precipitated under these conditions and was dialyzed instead against PBS supplemented with 0.3 M NaCl (0.45 M total NaCl) and 8% glycerol. The pH of the buffer (ranging from 7.5–8.5) had no obvious effect on protein solubility. The KCH_6 _protein was insoluble over a range of pH values and salt concentrations (up to 0.8 M), and the protein was not evaluated further. The remaining proteins could be prepared at final concentrations above 1 mg/ml except HNCM, which precipitated out of solution at protein concentrations above 500 μg/ml. The specific activities of the tagged fusion proteins were similar, ranging from 4.3–8.5 × 10^4 ^U/mg protein, corresponding to 47–92% of the activity of the native enzyme (Figure 1).

### Native Cre recombinase has protein transduction activity that is enhanced by polyhistidine and NLS sequences

**Figure 2:**
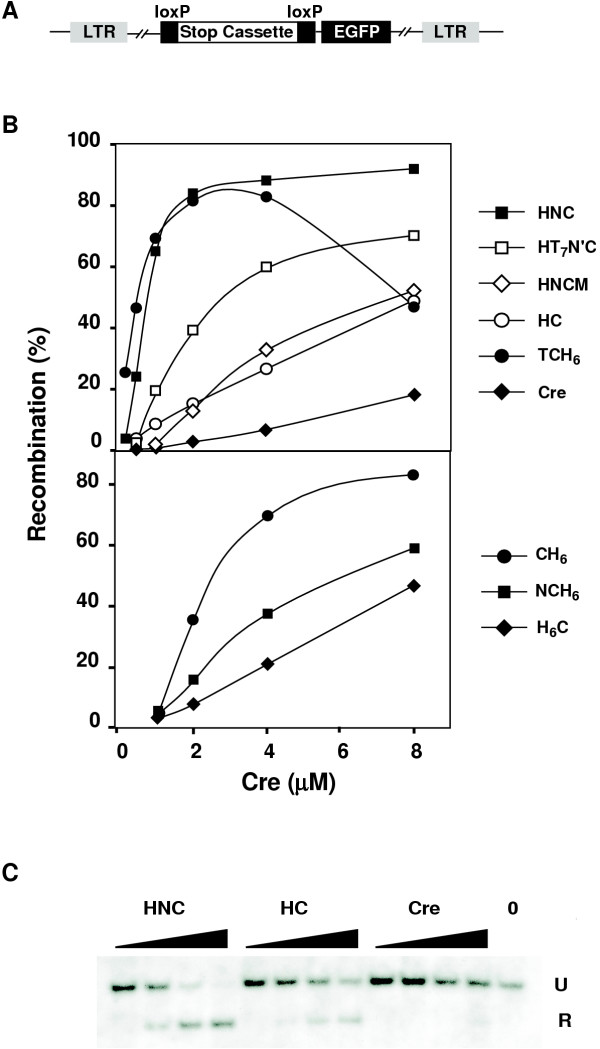
**Cre-mediated recombination in cultured cells. **(A). Structure of the recombination substrate in Tex.loxP.EG cells. The pBABE.lox.stp.EGFP retrovirus contains multiple polyadenylation sequences (stop cassette) flanked by loxP sites such that Cre-mediated recombination activates the expression of an enhanced green fluorescent protein (EGFP) reporter gene. (B). Dose-response of Cre-mediated recombination. Tex.loxp.EG cells were treated for two hours with increasing concentrations of each soluble Cre recombinase; were washed twice with PBS; and after 24 hours in normal growth medium, the percentage of GFP expressing cells was determined by FACS analysis. For clarity, the results obtained using different fusion proteins are plotted in two panels. The Upper Panel shows results for HNC (solid squares), HT7N'C (solid circles), HNCM (open squares), HC (open circles), and Cre (solid triangles). The Lower Panel plots the results for NCH_6 _(solid squares), H_6_C (solid circles), and CH_6 _(solid triangles). (C). Southern blot analysis of Cre-mediated recombination. Tex.loxp.EG cells were treated with 0.5, 1.0, 2.0, and 4.0 μM of HNC, HNCM and Cre proteins as described above, except after 24 hours DNA was extracted, digested with *Eco*R1 and analyzed by Southern blot analysis. Cre-mediated recombination results in the conversion of the loxP-containing fragment (upper band, U) to the recombination product (lower band, R).

The uptake and enzymatic activity of Cre proteins was monitored in Tex.loxP.EG cells [8]. These cells contain a single integrated retrovirus (Fig 2A) in which the expression of an enhanced green fluorescent protein (EGFP) gene is prevented by a "stop cassette" consisting of multiple polyadenylation sites positioned between two loxP sites. Deletion of the stop cassette by Cre mediated-recombination activates the expression of the EGFP reporter gene. Cells were exposed to Cre, HC, HNC, HT_7_N'C, HNCM, H_6_C, CH_6_, NCH_6_, and TCH_6 _for 2 hrs at concentrations ranging from 0 to 8 μM. The cells were then washed extensively with PBS, were cultured for 24 hrs to provide time for EGFP expression, and the percentage of EGFP-expressing cells was determined by flow cytometry. Recombination was also monitored by Southern blot hybridization (Fig 2C), thus confirming that expression of the EGFP reporter accurately reflected the extent of template recombination.

As shown in Figure 2B, all of the proteins tested induced recombination in a concentration-dependent manner. The native enzyme had the lowest activity, inducing recombination in up to 17% of the cells. Uptake and/or activity was increased by polyhistidine tags positioned on either the amino- (HC and H_6_C) or carboxy-terminal (CH_6_) ends of the enzyme. HC, which contains the 6xHis tag from pET28a(+), and H_6_C and CH_6_, which contain simple 6xHis sequences, induced similar levels of recombination, ranging between 45 and 60% of cells. Activity was further increased by the addition of an SV40 large T antigen nuclear localization signal (HNC, HT_7_N'C and NCH). At low concentrations, TCH_6 _was the most active protein tested, but higher levels of the protein were toxic to cells, presumably because of the concentration of NaCl required to maintain protein solubility during preparation of the enzyme. Finally, presence of the FGF-4 MTS sequence proved inhibitory for recombination in cultured cells. HNC, which lacks the MTS sequence, was approximately 8 times more active than HNCM as determined by the concentration of enzyme required to induce recombination in 50% of the cells. Thus, while the FGF-4 MTS has been used to transduce a variety of proteins and peptides into mammalian cells, the sequence suppressed the activity of cell-permeant Cre. This confirms the results of a earlier study, reported while this work was in progress [10].

### Temperature-dependent protein transduction

**Figure 3:**
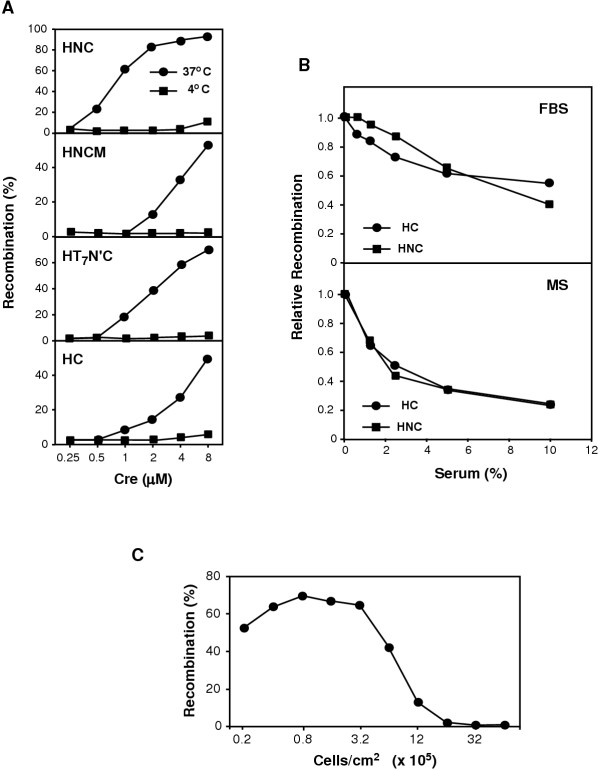
**Temperature, serum and cell density effects on Cre-mediated recombination. **(A) Transduction of cell-permeant Cre is inhibited at 4°C. Tex.loxp.EG cells were treated for 2 hours with increasing concentrations of the indicated enzymes at 4 or 37°C and the cells were processed and analyzed for Cre-mediated recombination as described in Figure 1. (B) Effect of serum on Cre-mediated recombination. Tex.loxp.EGFP cells were treated for 2 hours with 2 μM HNC or with 5 μM HC in the presence of increasing concentrations of fetal bovine serum (FBS) or normal mouse serum (MS) and analyzed for Cre-mediated recombination as described in Figure 1. The extent of recombination (percent GFP positive cells) was normalized to cells treated in the absence of serum. (B) Effect of cell density on Cre-mediated recombination. Increasing concentrations of Tex.loxp.EGFP cells were treated for 2 hours with 2 μM HNC and were analyzed for Cre-mediated recombination as described in Figure 1.

Transduction of cargoes containing the FGF-4 MTS [17], including the HNCM protein used in this study [25], is greatly decreased at 4°C as compared to 37°C. By contrast, there have been conflicting reports with regard to the transduction of proteins containing the Tat and Antennapedia PTDs [26-33]. Cre fusion proteins with (HNCM) and without (HNC, HT_7_N'C and HC) the FGF-4 MTS were therefore tested for their ability to enter cells at 4°C (Fig 3A). Tex.loxP.EG cells were incubated with varying concentrations of the proteins for one hour at either 37°C or 4°C; were washed extensively and were cultured at 37°C to allow time for EGFP expression. In each case, the uptake of the enzyme was inhibited at 4°C, indicating that the inhibitory effects of low temperature are not limited to cargoes containing the FGF-4 MTS. Low levels of recombination observed in cells treated with higher concentrations probably results from cell-associated enzyme that the washing steps fail to remove and that gains entry when cells are later cultured at 37°C.

### Serum and cell density effects on protein transduction

Serum has been reported to inhibit the transduction of cargoes containing the FGF-4 MTS; whereas, transduction of proteins containing the Tat and Antennapedia PTDs appears to be unaffected by serum. The effects of serum on protein transduction were assessed by treating TexloxP.EG cells with either HC (5 μM) or HNC (2 μM) for 1 hour in the presence of varying concentrations of either fetal bovine serum (Figure 3B, FBS) or normal mouse serum (Figure 3B, MS). Recombination induced by both proteins was inhibited by up to 60% and 80% in media containing 10% FBS and MS, respectively. Serum appeared to inhibit protein transduction specifically, since it had no discernable effect on either the stability or activity of the proteins *in vitro *(data not shown). These results indicate that the inhibition of protein transduction by serum is not limited to cargoes containing the FGF-4 MTS.

To assess the effects of cell density on protein transduction, TexloxP.EG cells were seeded at different concentrations in 2 cm^2 ^culture dishes and were treated with HNC (2 μM) for 1 hour (Figure 3C). Recombination efficiencies increased by about 40% as the number of number of cells was increased from 10^4 ^to 6 × 10^4 ^cells/cm^2^, and then declined sharply at concentrations above 2 × 10^5 ^cells/cm^2^. Tex.loxP.EG is a T-cell line and is non-adherent; however, the cells settle to the bottom of the culture dish. The optimum density for recombination was similar to the number of cells (.75 × 10^4 ^cells/cm^2^) required to cover the culture dish.

### Uptake and localization of cell-permeant Cre proteins

**Figure 4:**
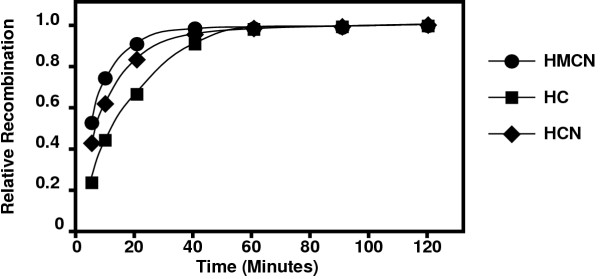
**Kinetics of Cre binding and/or uptake. **Tex.loxp.EGFP cells were treated with HNC (3 μM), HNCM (10 μM) and HC (5 μM) for different lengths of time; were washed three times with PBS and were analyzed for Cre-mediated recombination as described in Figure 1.

The kinetics of cell-permeant Cre uptake in cultured cells was monitored by examining cells for recombination after exposure to cell-permeant Cre for different lengths of time (Figure 4). Cells were treated with HC (5 μM), HNC (3 μM) and HNCM (10 μM) for 5 to 120 mins, were washed extensively and the extent of recombination was monitored 24 hours later by flow cytometry. Thus, the assay measures the time required for extracellular enzyme to become committed to cell entry. The uptake of HC, HNC and HNCM increased with time, reaching half-maximum levels after 15–20 min. Recombination was induced in cells following exposure to HNC and HNCM proteins for less time than to HC, suggesting a direct role for the NLS in promoting cell binding and/or entry. Note that the observed differences (apparent within minutes after exposing cells to Cre) cannot reflect potential differences in nuclear trafficking since a delay in nuclear import of a few minutes would not affect the extent of recombination measured 24 hours later.

**Figure 5:**
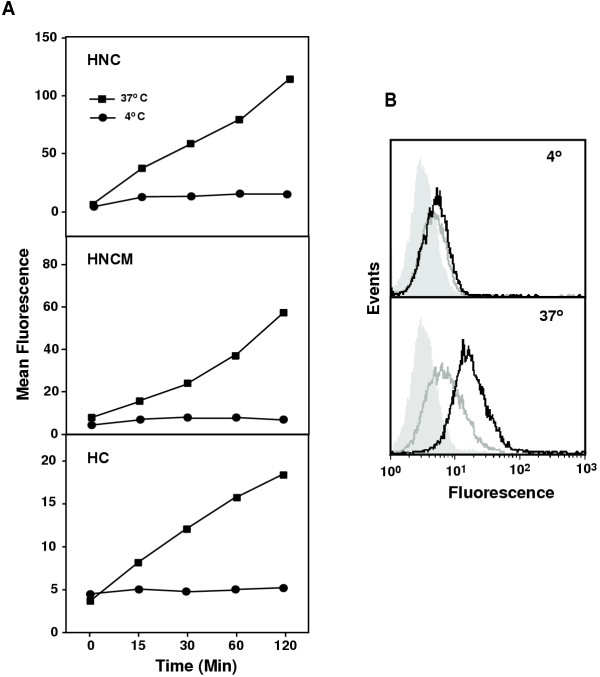
**Uptake of fluorescent Cre fusion proteins. **Tex.loxp.EG cells were treated for different lengths of time with the indicated Alexa 488-labeled Cre fusion proteins at 4 or 37°C and protein uptake was monitored by flow cytometry. (A) Kinetics of protein uptake. The mean fluorescence of cells treated with Alexa 488-labeled Cre fusion proteins, as calculated by the CellQuest software, increased with time and was inhibited at 4°C. (B) Representative FACS profiles of cells treated with the fluorescent HC protein. Cells were treated with Alexa 488-labeled HNCM at the indicated temperature for 0 (light gray, solid), 15 (medium gray) and 120 min (black) and analyzed by flow cytometry. Profiles of cells treated for 30 and 60 min. have been omitted for clarity.

**Figure 6:**
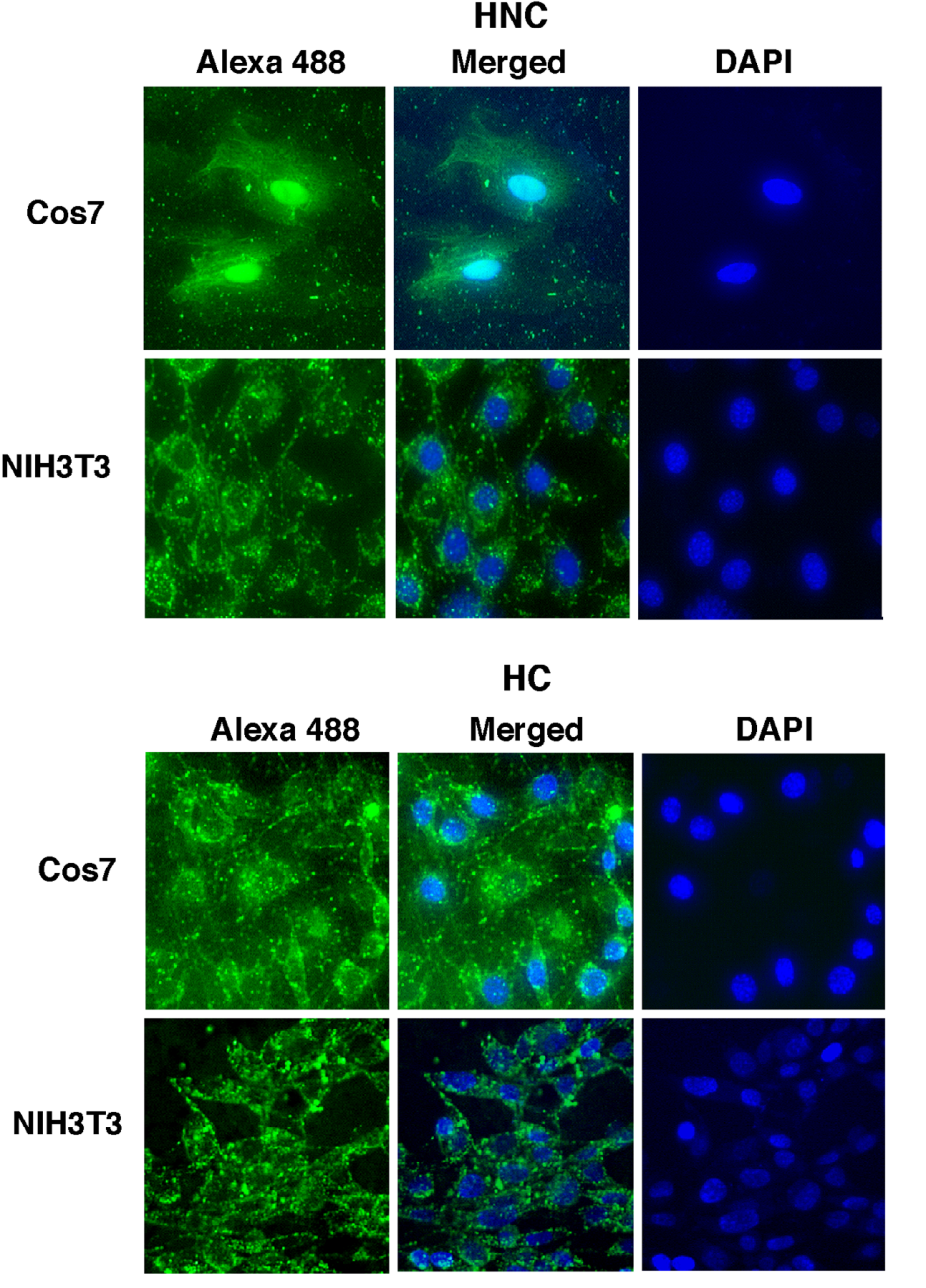
**Intracellular localization of fluorescent Cre fusion proteins. **Alexa 488-labeled HNC or HC proteins (1 μM) were incubated with Cos7 or NIH3T3 cells for 1.5 hr, and the living cells were directly visualized by fluorescence microscopy (green fluorescence, left panels). Cell nuclei counter stained with DAPI (blue fluorescence) are shown in the right panels and the merged images are shown in the middle panels.

The uptake and localization of cell-permeant Cre in cultured cells was also monitored in living cells by using proteins labeled with the fluorescent dye, Alexa 488 (Figures 5 and 6). The uptake of tagged HC, HNC and HNCM increased with the time as measured by flow cytometry (Figure 5). Again, HNC and HNCM appeared to enter cells more rapidly than HC, which lacks an NLS. Note that the magnitude of the fluorescence is less important than the rate of increase (slope) since the proteins were not labelled to the same extent with Alexa 488.

The localization of Alexa 488-tagged HNC and HC proteins was monitored in living cells by fluorescence microscopy. The HNC protein was localized to the nuclei of Cos7 cells, but was predominately cytoplasmic in NIH3T3 cells (Fig 6B). Cre protein was also localized to the nuclei of Tex.loxP.EGcells (data not shown).

### Recombination in different cell types

**Figure 7:**
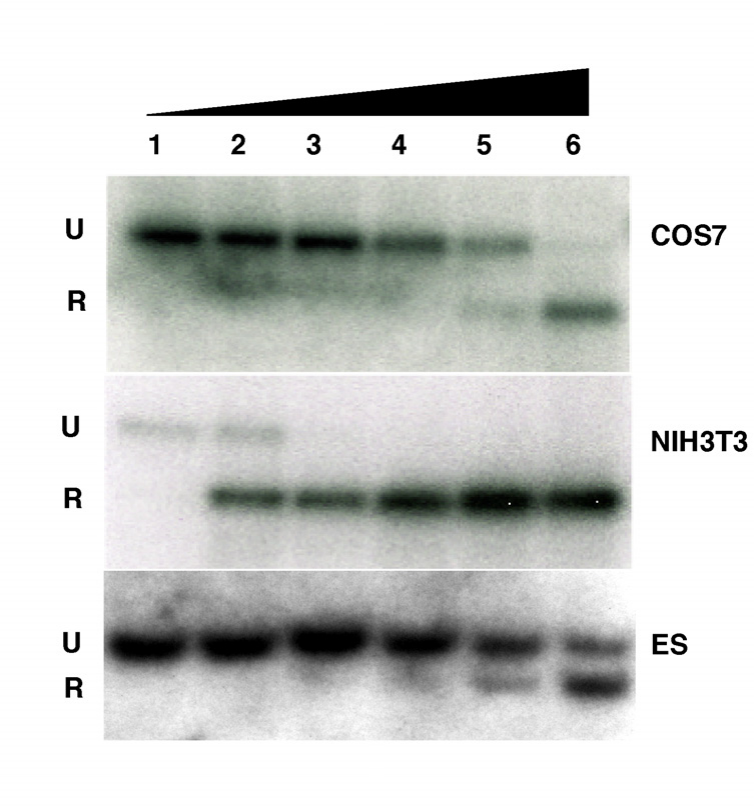
**Cre-mediated recombination in different cell types. **Cos7 and NIH3T3 cells containing a single integrated copy of the pBABE.lox.stp.EGFP retrovirus and murine embryonic stem (ES) cells containing a floxed IKK γ gene were treated with 0, 0.5, 1.0, 2.0, 5.0, and 10 μM HNC (Cos7 and NIH3T3) or with 0, 0.25, 0.5, 1.0, 2.0, and 5.0 μM HNC (ES cells) for two hours. Cellular DNA was extracted, digested with *Eco*RI, and analyzed by Southern blot analysis. Cre-mediated recombination results in the conversion of the loxP-containing fragment (upper band, U) to the recombination product (lower band, R).

**Figure 8:**
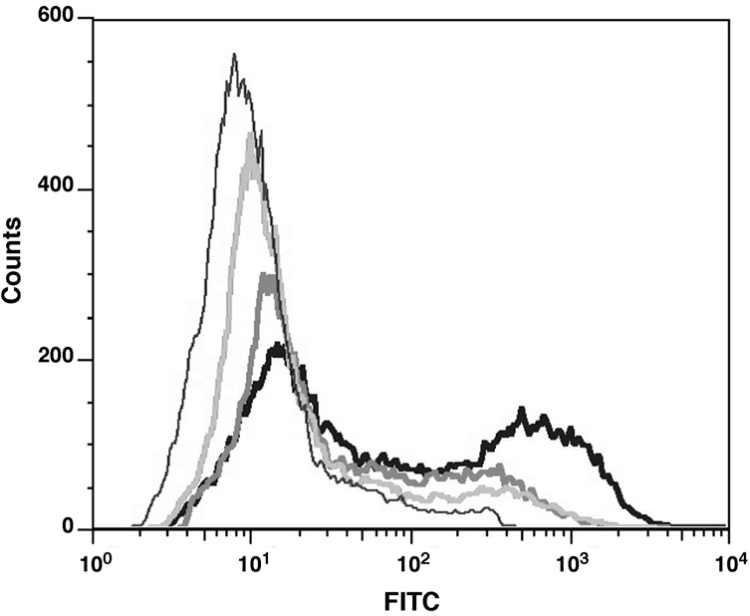
**Cre-mediated recombination in primary splenocytes. **Splenocytes from ROSA-26R mice were treated with 0 (thin black line), 1.0 (light grey line), 2.0 (dark grey line) and 5.0 (thick black line) μM HNC recombinase in serum free media for 2 hours. The cells were washed, cultured for 24 hours, stained with a fluorescent β-galactosidase substrate (ImaGene green™, C_12_FDG) and analyzed by flow cytometry. Cre-mediated recombination, which activates the expression of a floxed *lacZ *gene, results in increased fluorescence.

Several mammalian cell types were analyzed for their ability to undergo Cre-mediated recombination. Clones of Cos7 and NIH3T3 cells containing a single pBABE.lox.stp.EGFP provirus were treated with HNC for two hour and analyzed by Southern blot hybridization (Figure 7). Although the concentration of enzyme necessary to achieve nearly complete recombination was approximately 10 times higher in Cos7 cells than in NIH3T3 cells (10 versus 1 μM, Figure 7), the recombination efficiency did not correlate with localization of the enzyme to the nucleus (Figure 6). Extensive recombination was also observed in murine embryonic stem cells containing a floxed IKK γ gene (Figure 7) and in primary splenocytes explanted from ROSA26R mice (Figure 8). Efficient recombination was therefore observed in all mammalian cell types examined.

## Discussion

Cell permeant Cre proteins have generated considerable interest as genetic tools to regulate gene structure and function in mammalian cells [5]. In addition, Cre mediated recombination provides a quantitative reporter for studies on the protein transduction process itself. In the present study, 11 recombinant fusion proteins were analyzed to characterize sequences and conditions that affect protein uptake and/or activity and to develop more active cell-permeant enzymes. We report that the native enzyme has a low, but intrinsic ability to enter cells. Uptake and was enhanced by the addition of a 6xhistidine-tag on either the amino or carboxyl terminal ends of the protein and was enhanced further by the addition of a nuclear localization sequence from SV40 large T antigen or the transduction sequence from the HIV Tat protein. Finally, the hydrophobic membrane translocation sequence (MTS) from fibroblast growth factor 4 (FGF-4) had a net deleterious effect on Cre-mediated recombination in cultured cells.

We had hoped that the native or 6xHis-tagged enzymes would lack transduction activity so that the effects of additional sequences on cell entry, nuclear transport and recombination could be compared. However, we found that the native Cre protein has an intrinsic ability to enter cells, thus confirming observations by Will, et al. [11]. The mechanism by which Cre gains entry into cells remains to be determined. The enzyme may possess a protein transduction domain analogous to those described for HIV Tat, Antennapedia and a growing list of proteins that can enter cells directly, without requiring specific receptor and transporter systems [34]. The fact that Cre is a basic protein [23] is potentially significant considering that many basic peptides are capable of entering cells [24,35-38].

The transduction activity of the native enzyme hinders quantitative studies of sequences incorporated into recombinant Cre proteins, since structural changes associated with each modification may have varying effects on the intrinsic ability of Cre to enter cells. Even so, the 6xHis sequence appeared to facilitate cell entry, since two different 6xHis sequences enhanced Cre-mediated recombination while having little effect on the activity of the enzyme *in vitro*. Moreover, 6xHis sequences were active when positioned on either the amino- or carboxyl-terminal ends of the enzyme. L-histidine heptamers have been shown to enter cells, although much less efficiently than arginine homopolymers [37,38]. Positively charged histidine sequences also bind cell surface heparin sulfate proteoglycans [39,40], and thus may enhance uptake as has been suggested for the HIV Tat transduction sequence [41]. The transduction activity of the 6xHis sequences is potentially significant given their widespread use as affinity tags to purify recombinant fusion proteins [22].

We [8] and others [10] have shown that the activity of cell-permeant Cre fusion proteins in cultured cells can be enhanced by the addition of an SV40 large T antigen nuclear localization sequence (NLS). The NLS has been shown to enhance the activity of Cre expression vectors [42], presumably by targeting the protein to the nucleus. However, in the present study the NLS enhanced the delivery of Cre fusion proteins into cultured cells as assessed either by cell-based recombination or by uptake of fluorescent Cre proteins. Moreover, nuclear localization did not appear to contribute to cell type-specific differences in the activity of the HNC protein. These results are consistent with the observation that the T antigen NLS, like a number of other basic sequences, has protein transduction activity [24].

HNC, which consisted of a 6xHis tag and T antigen NLS attached to the amino-terminus of the enzyme, was highly active despite the absence of a canonical protein transduction sequence. The 6xHis tag and NLS sequences separately contributed to the transduction of the enzyme, which was 5–8 times more active in cultured cells than the HisNLSCreMTS (HNCM) protein described in our earlier study [8]. By virtue of their positive charge, these elements share similarities with basic protein transduction domains such as HIV Tat and Antennapedia. Early studies suggested that the basic PTDs entered cells through an energy-independent process that was relatively unaffected by low (4°C) temperature [26-30]. However, later studies suggest that the positively charged PTDs promote cell uptake by endocytosis possibly by binding negatively charged proteins on the cell surface [12,31-33,43,44]. Similarly, the uptake of Cre fusion proteins is consistent with an endocytic mechanism. In particular, uptake (time required for commitment to entry) was relatively rapid (10 to 15 min for half maximum uptake), was greatly decreased at 4°C, and was inhibited by up to 80% by serum. The inhibition by serum appeared to involve cell binding and/or entry specifically, since serum had no discernable effect on the stability or activity of Cre fusion proteins *in vitro *(data not shown). Recombination was markedly suppressed at higher cell densities, possibility because binding sites in or on cells sequester the enzyme, thus lowering the effective protein concentration. Alternatively, cell density may suppress protein transduction by reducing cell size and/or available surface area [25]. In either event, similar observations have been reported for a TatCre protein that lacks a 6xHis tag [9], illustrating the need to control for cell density when comparing the effects or cell-permeant proteins in different cell populations.

## Conclusions

The effects of different sequences on Cre uptake or activity were difficult to predict in advance, and consequently, the process of developing a more active cell-permeant recombinase was largely empirical. Proteins containing amino-terminal Tat and (KFF)_3_K sequences (TCH_6 _and KCH_6_) were poorly soluble when dialyzed against isotonic media, and the FGF-4 MTS interfered with the activity of the enzyme in cultured cells. Since Cre is probably not unique in this regard, investigators seeking to develop other cell-permeant proteins would be advised to test a variety of sequences positioned at different places on the protein. In the present case, the HNC protein possesses an outstanding combination of activity, solubility and yield that will enhance the use of cell-permeant Cre to regulate gene structure and function in living cells.

## Methods

### Cre expression vectors

Native Cre and Cre fusion proteins were expressed in *E. coli *from pET28a (+)-based plasmids (Novagen). A plasmid expressing native Cre (Cre) was constructed by using the Cre #1 and Cre-stop-HindIII primers to amplify Cre coding sequences from MBP-NLS-Cre-MTS [8] which were cloned between the *Nco*I and *Hind*III sites of pET28a (+). HC, His_6_C, HNC, and HT_7_C were similarly constructed by PCR amplification, using primers Cre #2, Cre #3, Cre #4 and Cre #5, respectively, together with Cre-stop-HindIII primer. The amplified DNA fragments were cloned into *Nhe*I and *Hind*III sites of pET28a (+). CHis_6_, NCHis_6_, MCHis_6_, TATCHis_6_, and (KFF)_3_KCHis_6 _were constructed by using primers Cre #6, Cre #7, Cre #8, Cre #9 and Cre #10, respectively, together with the Cre-XhoI primer, and then cloned into the *Nco*I and *Xho*I sites of pET28b(+).

Cre #1: AGAGAGCCATGGGCTCCAATTTACTGACCGTACACCAA

Cre #2: GTACATGCTAGCTCCAATTTACTGACCGTACACCAA

Cre#3: AGAGAGCCATGGGCCATCATCATCATCATCACAGCTCCAATTTACTGACCGTACACCAA

Cre#4: GTACATGCTAGCCCAAGAAGAAGAGGAAGGTGCTCCAATTTACTGACCGTACACCAA

Cre#5: GTACATGAATTCTCCAATTTACTGACCGTACACCAA

Cre#6: AGAGAGCCATGGGCTCCAATTTACTGACCGTACACCAA

Cre#7: AGAGAGCCATGGGCCCCAAGAAGAAGAGGAAGGTGTCCAATTTACTGACCGTACACCAA

Cre#8: AGAGAGCCATGGGCGCAGCCGTTCTTCTCCCTGTTCTTCTTGCCGCACCCTCCAATTTACTGACCGTACACCAA

Cre#9: AGAGAGCCATGGGCGGTCGCAAGAAACGTCGCCAACGTCGCCGTTCCAATTTACTGACCGTACACCAA

Cre #10: AGAGAGCCATGGGCAAATTCTTTAAGTTCTTTAAGTTCTTTAAGTCCAATTTACTGACCGTACACCAA

Cre-stop-HindIII: GATACGAAGCTTCTACTAATCGCCATCTTCCAGCAGGCGC

Cre – XhoI: AGAGAGCTCGAGATCGCCATCTTCCAGCAGGCGCACCATTGCCCCTGT

### Protein purification

Polyhistidine-tagged Cre fusion proteins were expressed in *E. coli *strain BL21 (DE3) and purified by Ni^2+ ^affinity chromatography as described previously [8]. Bacterial cultures (2L) were grown to an A_600 _of 0.6–1.0, were induced with 0.4 mM IPTG, and after harvesting the cells were lysed in 40 ml 50 mM Tris (pH 8.0), 50 mM sodium phosphate and 300 mM NaCl. After affinity chromatography, recombinant proteins were dialyzed against DMEM or RPMI media, except TCH_6 _and KCH_6_, which precipitated under these conditions. TCH_6 _was dialyzed instead against PBS supplemented with 0.3 M NaCl (0.45 M NaCl final) and 8% glycerol. The native recombinase was similarly expressed; however, lysates were applied to a 50 ml hydroxyapatite column and step-eluted with sodium phosphate buffers (pH 8.0) of increasing concentration (50, 100, 200, 300, and 500 μM). The peak fraction (200 μM) was dialyzed against PBS, was concentrated by centrifugal ultra filtration to about 20 mg/ml and was fractionated by Sephacryl S-100 HR gel filtration FPLC. Peak fractions identified by SDS PAGE, were concentrated and dialyzed against PRMI-1640 medium containing 1% streptomycin/penicillin and 2% glycerol.

*In vitro *assays of Cre enzyme activity measured the release of a circular plasmid inserted into a λ phage (Novagen, Madison, WI) by transformation of *E. coli *[8]. One unit (U) of enzyme produces 10^4 ^colonies (equivalent to 2 × 10^6 ^circular molecules) in a 30 minute reaction containing 200 ng DNA substrate in 50 mM Tris HCl, pH 7.5, 33 mM NaCl and 10 mM MgCl_2 _in a total volume of 15 μl. All Cre proteins were stable for at least 6 months at -80°C without significant loss of enzymatic activity.

### Cell culture and protein transduction

Tex.loxp.EG, 3T3.loxp.EG and Cos7.loxp.EG cells were derived from Tex (a murine thymoma line derived from p53-deficient mice), NIH3T3 and Cos7 cells, respectively, following infection with the pBABE.lox.stp.EGFP retrovirus [8]. Cells were incubated with serum-free RPMI 1640 (Tex.loxp.EG) or DMEM (3T3.loxp.EG and Cos7.loxp.EG) containing Cre fusion proteins for 2 hours, then washed with PBS twice and cultured in normal growth medium at 37°C incubator for 24 hours. Cre mediated recombination, which induces the expression of an enhanced green fluorescence protein (EGFP) in Tex.loxp.EG cells, was measured by flow cytometry using a FACSort instrument (Becton Dickinson). Alternatively, recombination was monitored by Southern blot hybridization [8]. For experiments involving low temperature protein transduction, the cells and all solutions were maintained at 4°C until after washing with cold PBS, after which the cells were returned to normal medium at 37°C.

### Preparation of fluorescent HNC, HC, and HNCM proteins

Fluorescent HNC, HC, and HNCM proteins were prepared by using the Alexa 488 protein labeling kit (Molecular Probes, catalog number A-10235), according to the manufacturer's instructions. The proteins were dialyzed against PBS, were labeled at a concentration of 2 mg/ml and purified by gel filtration through a Sephadex G50 column. Cells were treated with the fluorescent proteins at a final concentration of 1 μM.

The localization of fluorescent Cre proteins was monitored in living NIH3T3 and Cos7 cells by fluorescence microscopy. The cells were cultured to 50–80% confluence in a slide chamber (NUNC), washed with PBS and then incubated with fluorescent Cre proteins at a final concentration of 1 μM for 1.5 hours. The cells were washed three times with PBS, counterstained with 1 μg/ml 4',6-diamidino-2-phenylindole (DAPI) in PBS for 20 minutes, washed three times with PBS and mounted in anti-fade fluorescence mounting medium. The stained cells were photographed with an Olympus BX60 fluorescence microscope using green and blue filters.

### Ex vivo recombination by HNC recombinase in primary cells

Splenocytes from ROSA-26R mice were cultured for one day in RPMI 1640 medium containing 10% FBS and were treated with HNC recombinase in serum free media for 2 hours. The cells were washed, cultured for 24 hours, and stained with a fluorescent β-galactosidase substrate (ImaGene green™, C_12_FDG) according to instructions provided by the manufacturer (Molecular Probes, Inc.) Recombination, which activates the expression of a floxed *lacZ *gene, was assessed by flow cytometry.

## Authors' contributions

QL developed and characterized most of the recombinant Cre proteins described in this study. DJ provided the HNCM protein and assisted with in vitro assays for Cre activity. KDGA. investigated inhibition of Cre activity by serum. ER supervised the project and drafted the final manuscript. All authors approved the final manuscript.

## References

[B1] Nagy A (2000). Cre recombinase: the universal reagent for genome tailoring. Genesis.

[B2] Kuhn R, Schwenk F, Aguet M, Rajewsky K (1995). Inducible gene targeting in mice. Science.

[B3] Yu Y, Bradley A (2001). Engineering chromosomal rearrangements in mice. Nat Rev Genet.

[B4] Lewandoski M (2001). Conditional control of gene expression in the mouse. Nat Rev Genet.

[B5] Chen CM, Behringer RR (2001). CREating breakthroughs. Nat Biotechnol.

[B6] Silver DP, Livingston DM (2001). Self-excising retroviral vectors encoding the Cre recombinase overcome Cre-mediated cellular toxicity. Mol Cell.

[B7] Loonstra A, Vooijs M, Beverloo HB, Allak BA, van Drunen E, Kanaar R, Berns A, Jonkers J (2001). Growth inhibition and DNA damage induced by Cre recombinase in mammalian cells. Proc Natl Acad Sci U S A.

[B8] Jo D, Nashabi A, Doxsee C, Lin Q, Unutmaz D, Chen J, Ruley HE (2001). Epigenetic regulation of gene structure and function with a cell- permeable Cre recombinase. Nat Biotechnol.

[B9] Joshi SK, Hashimoto K, Koni PA (2002). Induced DNA recombination by Cre recombinase protein transduction. Genesis.

[B10] Peitz M, Pfannkuche K, Rajewsky K, Edenhofer F (2002). Ability of the hydrophobic FGF and basic TAT peptides to promote cellular uptake of recombinant Cre recombinase: a tool for efficient genetic engineering of mammalian genomes. Proc Natl Acad Sci U S A.

[B11] Will E, Klump H, Heffner N, Schwieger M, Schiedlmeier B, Ostertag W, Baum C, Stocking C (2002). Unmodified Cre recombinase crosses the membrane. Nucleic Acids Res.

[B12] Wadia JS, Stan RV, Dowdy SF (2004). Transducible TAT-HA fusogenic peptide enhances escape of TAT-fusion proteins after lipid raft macropinocytosis. Nat Med.

[B13] Dunican DJ, Doherty P (2001). Designing cell-permeant phosphopeptides to modulate intracellular signaling pathways. Biopolymers.

[B14] Hawiger J (1999). Noninvasive intracellular delivery of functional peptides and proteins. Curr Opin Chem Biol.

[B15] Schwarze SR, Hruska KA, Dowdy SF (2000). Protein transduction: unrestricted delivery into all cells?. Trends Cell Biol.

[B16] Lundberg M, Johansson M (2001). Is VP22 nuclear homing an artifact?. Nat Biotechnol.

[B17] Lin YZ, Yao SY, Veach RA, Torgerson TR, Hawiger J (1995). Inhibition of nuclear translocation of transcription factor NF-kappa B by a synthetic peptide containing a cell membrane-permeable motif and nuclear localization sequence. J Biol Chem.

[B18] Frankel AD, Pabo CO (1988). Cellular uptake of the tat protein from human immunodeficiency virus. Cell.

[B19] Good L, Awasthi SK, Dryselius R, Larsson O, Nielsen PE (2001). Bactericidal antisense effects of peptide-PNA conjugates. Nat Biotechnol.

[B20] Kalderon D, Roberts BL, Richardson WD, Smith AE (1984). A short amino acid sequence able to specify nuclear location. Cell.

[B21] Le Y, Gagneten S, Tombaccini D, Bethke B, Sauer B (1999). Nuclear targeting determinants of the phage P1 cre DNA recombinase. Nucleic Acids Res.

[B22] Gaberc-Porekar V, Menart V (2001). Perspectives of immobilized-metal affinity chromatography. J Biochem Biophys Methods.

[B23] Sternberg N, Sauer B, Hoess R (1986). Bacteriophage P1 cre gene and its regulatory region. Evidence for multiple promoters and for regulation by DNA methylation. J Mol Biol.

[B24] Futaki S, Suzuki T, Ohashi W, Yagami T, Tanaka S, Ueda K, Sugiura Y (2001). Arginine-rich peptides. An abundant source of membrane-permeable peptides having potential as carriers for intracellular protein delivery. J Biol Chem.

[B25] Jo D, Lin Q, Nashabi A, Mays DJ, Unutmaz D, Pietenpol JA, Ruley HE (2003). Cell cycle-dependent transduction of cell-permeant Cre recombinase proteins. J Cell Biochem.

[B26] Derossi D, Joliot AH, Chassaing G, Prochiantz A (1994). The third helix of the Antennapedia homeodomain translocates through biological membranes. J Biol Chem.

[B27] Elliott G, O'Hare P (1997). Intercellular trafficking and protein delivery by a herpesvirus structural protein. Cell.

[B28] Mann DA, Frankel AD (1991). Endocytosis and targeting of exogenous HIV-1 Tat protein. EMBO J.

[B29] Morris MC, Depollier J, Mery J, Heitz F, Divita G (2001). A peptide carrier for the delivery of biologically active proteins into mammalian cells. Nat Biotechnol.

[B30] Morris MC, Vidal P, Chaloin L, Heitz F, Divita G (1997). A new peptide vector for efficient delivery of oligonucleotides into mammalian cells. Nucleic Acids Res.

[B31] Console S, Marty C, Garcia-Echeverria C, Schwendener R, Ballmer-Hofer K (2003). Antennapedia and HIV transactivator of transcription (TAT) "protein transduction domains" promote endocytosis of high molecular weight cargo upon binding to cell surface glycosaminoglycans. J Biol Chem.

[B32] Richard JP, Melikov K, Vives E, Ramos C, Verbeure B, Gait MJ, Chernomordik LV, Lebleu B (2003). Cell-penetrating peptides. A reevaluation of the mechanism of cellular uptake. J Biol Chem.

[B33] Fittipaldi A, Ferrari A, Zoppe M, Arcangeli C, Pellegrini V, Beltram F, Giacca M (2003). Cell membrane lipid rafts mediate caveolar endocytosis of HIV-1 Tat fusion proteins. J Biol Chem.

[B34] Prochiantz A (2000). Messenger proteins: homeoproteins, TAT and others. Curr Opin Cell Biol.

[B35] Mi Z, Mai J, Lu X, Robbins PD (2000). Characterization of a class of cationic peptides able to facilitate efficient protein transduction in vitro and in vivo. Mol Ther.

[B36] Ho A, Schwarze SR, Mermelstein SJ, Waksman G, Dowdy SF (2001). Synthetic protein transduction domains: enhanced transduction potential in vitro and in vivo. Cancer Res.

[B37] Mitchell DJ, Kim DT, Steinman L, Fathman CG, Rothbard JB (2000). Polyarginine enters cells more efficiently than other polycationic homopolymers. J Pept Res.

[B38] Wender PA, Mitchell DJ, Pattabiraman K, Pelkey ET, Steinman L, Rothbard JB (2000). The design, synthesis, and evaluation of molecules that enable or enhance cellular uptake: peptoid molecular transporters. Proc Natl Acad Sci U S A.

[B39] Hondal RJ, Ma S, Caprioli RM, Hill KE, Burk RF (2001). Heparin-binding histidine and lysine residues of rat selenoprotein P. J Biol Chem.

[B40] Lacy HM, Sanderson RD (2002). 6xHis promotes binding of a recombinant protein to heparan sulfate. Biotechniques.

[B41] Tyagi M, Rusnati M, Presta M, Giacca M (2001). Internalization of HIV-1 tat requires cell surface heparan sulfate proteoglycans. J Biol Chem.

[B42] Gu H, Zou YR, Rajewsky K (1993). Independent control of immunoglobulin switch recombination at individual switch regions evidenced through Cre-loxP-mediated gene targeting. Cell.

[B43] Drin G, Cottin S, Blanc E, Rees AR, Temsamani J (2003). Studies on the internalization mechanism of cationic cell-penetrating peptides. J Biol Chem.

[B44] Ziegler A, Blatter XL, Seelig A, Seelig J (2003). Protein transduction domains of HIV-1 and SIV TAT interact with charged lipid vesicles. Binding mechanism and thermodynamic analysis. Biochemistry.

